# Supplementary material for: Predicting visual function by interpreting a neuronal wiring diagram
Source: Nature. 2024 Oct 2;634(8032):113–23. doi: 10.1038/s41586-024-07953-5 (PMC11446822; doi:10.1038/s41586-024-07953-5)
Supplement: Supplementary file 6 — CRF and ERF predictions for individual TmY4 and TmY9 cells. Analogous to Supplementary Data 3, but for TmY target types. Shown are the top four monosynaptic pathways, the strongest pathway passing through each of the top ten intermediary types (ranking from Extended Data Fig. 7), and the trisynaptic pathway Tm1–TmY–Dm3–TmY (see the section entitled Prediction of spatial normalization). [file 41586_2024_7953_MOESM6_ESM.zip › DataS4/TmY9q/720575940639809880.pdf]

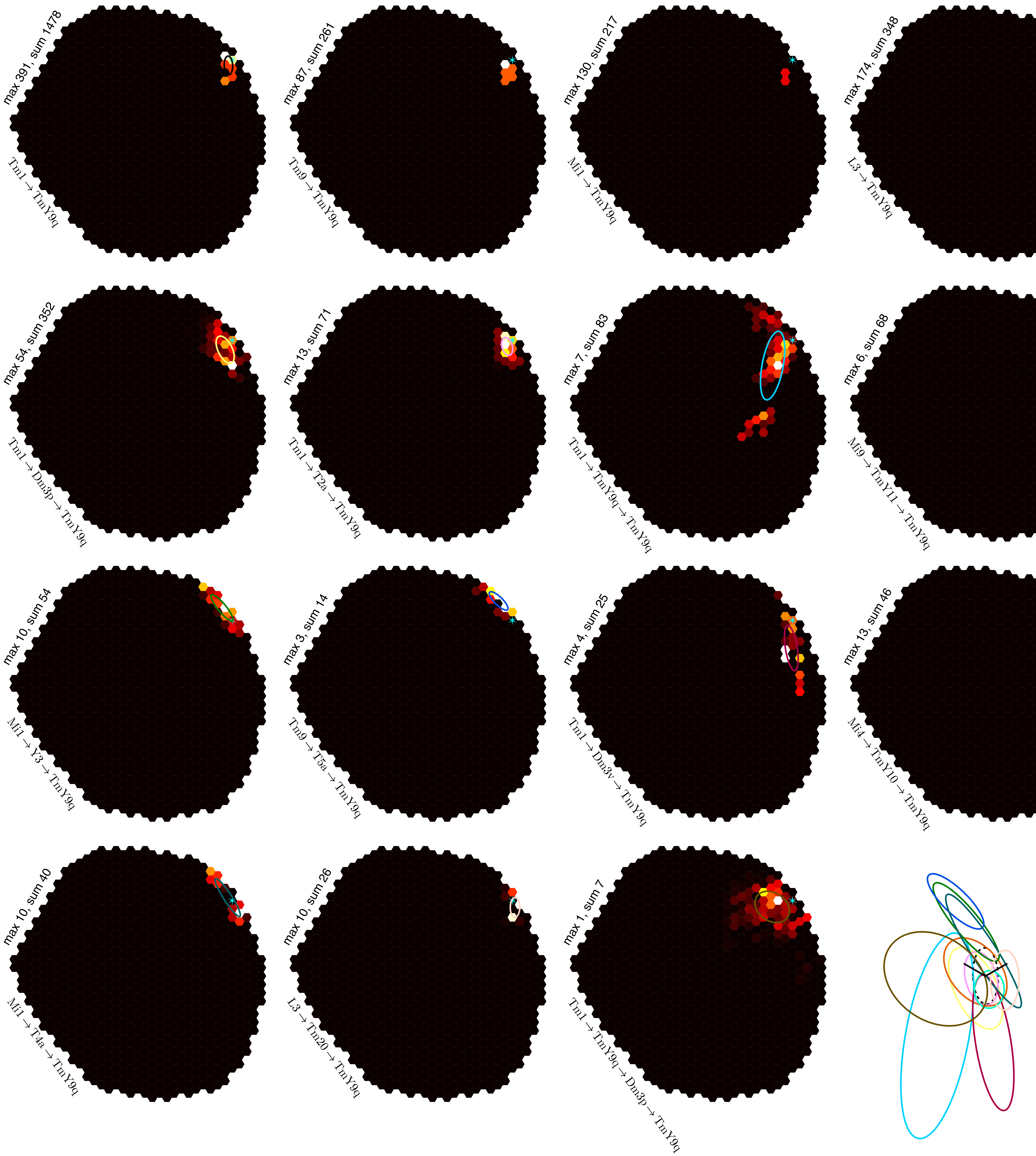

max 391, sum 1478
Tm1 → TmY9q
max 87, sum 261
Tm9 → TmY9q
max 130, sum 217
Mi1 → TmY9q
max 174, sum 348
L3 → TmY9q
max 54, sum 352
Tm1 → Dm3p → TmY9q
max 13, sum 71
Tm1 → T2a → TmY9q
max 7, sum 83
Tm1 → TmY9q → TmY9q
max 6, sum 68
Mi9 → TmY11 → TmY9q
max 10, sum 54
Mi1 → Y3 → TmY9q
max 3, sum 14
Tm9 → T5a → TmY9q
max 4, sum 25
Tm1 → Dm3v → TmY9q
max 13, sum 46
Mi4 → TmY10 → TmY9q
max 10, sum 40
Mi1 → T4a → TmY9q
max 10, sum 26
L3 → Tm20 → TmY9q
max 1, sum 7
Tm1 → TmY9q → Dm3p → TmY9q
